# Opto-Microfluidic System for Absorbance Measurements in Lithium Niobate Device Applied to pH Measurements

**DOI:** 10.3390/s20185366

**Published:** 2020-09-19

**Authors:** Riccardo Zamboni, Annamaria Zaltron, Elena Izzo, Gregorio Bottaro, Davide Ferraro, Cinzia Sada

**Affiliations:** 1 Physics and Astronomy Department G. Galileo, University of Padova, Via Marzolo 8, 35121 Padova, Italy; riccardo.zamboni@unipd.it (R.Z.); annamaria.zaltron@unipd.it (A.Z.); elena.izzo@studenti.unipd.it (E.I.); 2 Department of Chemistry, Institute of Condensed Matter Chemistry and Technologies for Energy (ICMATE), National Research Council (CNR), University of Padova, via Marzolo 1, 35131 Padova, Italy; gregorio.bottaro@cnr.it

**Keywords:** microfluidics, lithium niobate, integrated optical waveguides, optical measurements, pH sensor, lab-on-a-chip

## Abstract

The aim of Lab-on-a-chip systems is the downscaling of analytical protocols into microfluidic devices, including optical measurements. In this context, the growing interest of the scientific community in opto-microfluidic devices has fueled the development of new materials. Recently, lithium niobate has been presented as a promising material for this scope, thanks to its remarkable optical and physicochemical properties. Here, we present a novel microfluidic device realized starting from a lithium niobate crystal, combining engraved microfluidic channels with integrated and self-aligned optical waveguides. Notably, the proposed microfabrication strategy does not compromise the optical coupling between the waveguides and the microchannel, allowing one to measure the transmitted light through the liquid flowing in the channel. In addition, the device shows a high versatility in terms of the optical properties of the light source, such as wavelength and polarization. Finally, the developed opto-microfluidic system is successfully validated as a probe for real-time pH monitoring of the liquid flowing inside the microchannel, showing a high integrability and fast response.

## 1. Introduction

“Lab-On-a-Chip” (LOC) technology has nowadays become a stable means for the integration and miniaturization a wide range of standard chemical and biological analyses [1]. The advantages of these integrated microfluidics platforms are not only related to their portability, but also to their higher efficiency in terms of throughput and sample reagent consumption. Among others, one of the most promising aims of the LOC development is the realization of complex platforms, able to integrate on the same device several stages to perform a complete multi-step analysis [2,3,4,5]. In this framework, the material used for the realization of LOC systems plays a crucial role. The most common microfluidic devices are based on polymeric substrates—e.g., polydimethylsiloxane (PDMS), cyclic olefin copolymer (COC), and polymethylmethacrylate (PMMA)—due to their affordability and because they guarantee high flexibility in terms of design and fabrication [6,7]. Nevertheless, the integration of complex protocols could provide some issues in the miniaturization of multiple stages in these materials—for instance, the integration of optical probes. In fact, in this case, they are typically implemented in such devices by either the embedding of optical fibers [8,9,10,11,12] or by the realization of optical waveguides [13,14,15,16,17], using the polymer itself. However, the first solution shows the problem of reproducibility related to the alignment of the fibers with the other LOC components, leading each device to have different performances; meanwhile, the second still shows high light losses compared to the standard photonics materials due to their poor optical properties. An interesting solution to overcome these limitations could be represented by a material able to offer all the properties needed for the integration of different stages in the same substrate. For this aim, an interesting alternative to polymeric substrate is lithium niobate (LN), which can be worked as a glass substrate and presents a unique combination of optical and electrical properties. As a matter of a fact, in the field of integrated optics, LN is a well-known material for its properties that have been exploited for the realization of a wide range of optical devices, such as optical modulators [18], waveguides [19], second harmonic generators [20], photorefractive laser writing [21], or self-written waveguides [22]. Furthermore, very recently LN has been also demonstrated as a valuable substrate with which to realize microfluidic circuits, such as droplet generators [23,24,25]. Therefore, the combination of the optical capabilities typical of this material with microfluidic tools can offer unprecedented fertile ground for the development of advanced LOCs made using the same monolithic substrate. Additionally, during recent years LN has been also exploited for the realization of photovoltaic tweezers in iron-doped substrates, where photoinduced electric fields [26] were used to trap small particles [27]; to manipulate droplets [28], bubbles [29], or biological samples [30]; and to control liquid crystal cells [31,32,33,34]. Similarly, the pyroelectric effect of this material promotes creating local electric fields on its surface by means of temperature gradients, thus generating liquid microlenses [35], nano-pipetting [36], and 3D photorefractive red blood cell imaging [37]. Moreover, LN is also used for the realization of Surface Acoustic Waves (SAWs) in acoustofluidic devices [38,39,40,41,42]. Therefore, the potential capability of integrating all these properties into the same substrate makes LN a valuable material for realizing LOC systems. More in detail, the miniaturization of an optical probe across a microfluidic channel can represent a first fundamental building block for the subsequent realization of a more complex LOC system.

Herein, we propose a microfluidic device based on the miniaturized coupling between optical titanium waveguides and an engraved microfluidic channel, entirely in the same LN substrate. More in detail, a single straight waveguide is cut into two branches by the carving of the microchannel, thus giving rise to two perfectly aligned waveguides on the two sides of the channel. Therefore, the light injected into one branch (input waveguide) is guided across the channel, and then it is recoupled with the second waveguide branch (output waveguide). Notably, this microfabrication strategy not only provides a light transmission signal acting as probe of the optical properties of the liquid inside the channel, but also overcomes the alignment issues between the optical components and the fluidic one. Since the transmitted beam quality along the waveguide branches strongly depends on the mechanical cut of the LN substrate, we initially characterized the achievable microchannels using two different blades. Then, in order to verify the flexibility of this device, we tested different optical probes in terms of their wavelengths and polarization. Finally, as a proof of concept experiment, the LN-based optofluidic device is exploited as an effective sensor for optical pH detection, which is a key parameter to control in several LOC applications [43,44]. Notably, this application has been chosen considering that LN shows a high chemical resistance against acid/basic solutions. In detail, we start from two initial solutions prepared at pHs of 2.7 and 10.5, both spiked with Blue Bromothymol pH indicator. Then, a mixture of the two solutions is injected into an LN device and its transmittance, which varies according to its pH [45], is real-time monitored by the optical waveguide while flowing inside the microchannel.

## 2. Materials and Methods

### 2.1. Device Fabrication

The optofluidic device consists of two main parts: (i) the optical one, which is based on optical waveguides realized by titanium diffusion, and (ii) the fluidic microchannel. During the microfabrication process, firstly the waveguides are thermally diffused in a sample of lithium niobate (LN), and secondly the channel is engraved on the top of the crystal. This microfabrication procedure, which will be detailed in the following, not only is compatible with standard industrialized processes, but also guarantees an optimum self-alignment of the optical and fluidic components.

#### 2.1.1. Titanium Diffused Waveguides

**Figure 1:**
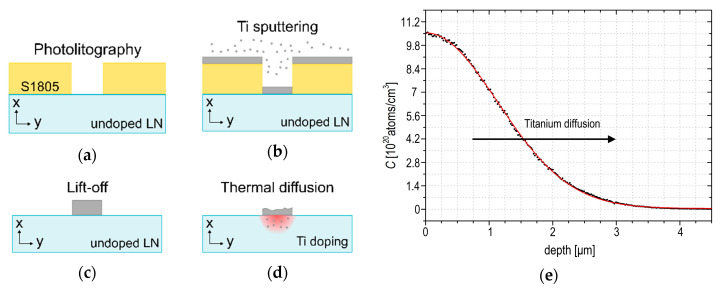
Titanium diffused channel waveguide fabrication on a commercial x-cut lithium niobate (LN) wafer. Sketches of the steps involved in the realization of the Ti-diffused waveguides, including (**a**) photolitography, (**b**) Ti deposition, (**c**) lift-off, (**d**) thermal diffusion. (**e**) The graph reports the profile of the titanium diffused inside x-cut lithium niobate, and the red line is a semi-gaussian interpolation of the data (black points).

Titanium (Ti) thermal diffusion by a deposited thin film is a conventional technique for the realization of channel waveguides in lithium niobate (Ti:LN) [19]. In fact, the titanium doping acts locally, changing the refractive index of the lithium niobate substrate, thus creating a zone where a light beam can be efficiently confined and guided. The waveguide realization consists of the following steps: photolithography, titanium deposition, lift-off, and subsequent thermal treatment, as shown in Figure 1a–d.

**Figure 2:**
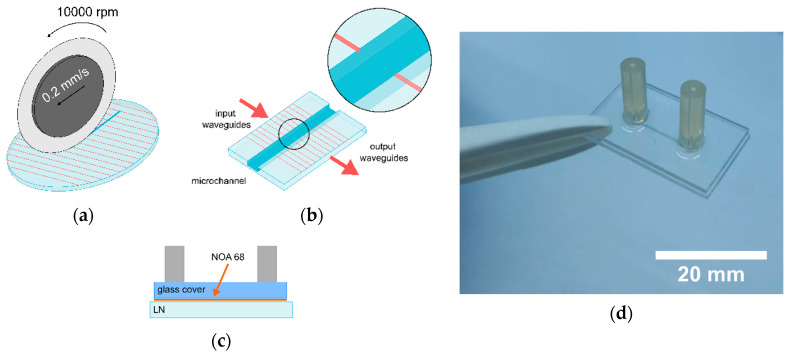
Schematic representation of the final steps realized to obtain the complete device. (**a**) The cutting technique allows one to engrave the microchannel and cut the samples from Ti:LN wafer. (**b**) The waveguides are divided by the microchannel in two branches, named input and output waveguides. The inset evidences the coupling of the waveguides and the channel. (**c**) The sample obtained from the Ti:LN is sealed with a glass cover and UV curable glue. (**d**) Picture of the final device.

Photolithography (Figure 1a) is performed onto a commercial x-cut wafer of lithium niobate (diameter: 3 inches; thickness: 1 mm, by Crystal Technology Inc., Palo Alto, CA, USA) using the photoresist S1805 (Dow Chemical, Midland, MI, USA), which is deposited by spin coating at 6000 rpm for 1 min following the datasheet guidelines. Before the UV exposure, a soft bake of the sample is performed at 80 °C for 15 min; the latter is the only procedure different from the guidelines, since lithium niobate may be subjected to crack formation if it is exposed to thermal shocking due to the pyroelectric effect. Therefore, a lower temperature than the value suggested by the guidelines (115 °C) is applied for a time long enough to assure the complete solvent evaporation. The exposure of the photoresist is performed for 13 s with an illumination intensity of 6.7 mW cm^−2^, and the development by MICROPOSIT MF-319 (Dow Chemical, Midland, MI, USA) lasts 1 min. Then, a 41 ± 5 nm Ti layer is deposited on the top of the wafer by magnetron sputtering (Figure 1b). After that, the lift-off process, allowing removing the residual photoresist (Figure 1c), is based on dipping the sample with photoresist and a Ti layer into a solution of SVC™-14 (Dow Chemical, Midland, MI, USA) for 20 min at 60 °C. In this way, the residual titanium deposited only on the photoresist is completely removed, thus leaving on the sample surface the titanium deposited directly on the LN surface. The sample is then cleaned from SVC™-14 by rinsing with isopropanol and distilled water. Finally, the 5 µm-wide Ti stripes are diffused into the underlying substrate by a thermal treatment performed inside a tubular furnace F-VS 100-500/13 (Carbolite Gero, Neuhausen, Germany) at 1030 °C for 2 h in an oxygen atmosphere (Figure 1d). The Secondary Ion Mass Spectrometry (SIMS) and Rutherford Back Scattering (RBS) techniques allow obtaining a complete characterization of the undiffused Ti concentration profile in the *x* direction of the crystal. As shown in Figure 1e, the in-depth dopant concentration follows a semi-gaussian profile, and the fit of the experimental data gives an estimation of the diffusion constant *D* = 89 ± 2 nm^2^ s^−1^, the mean diffused depth ω = (1.13 ± 0.05) µm, and the maximum Ti concentration *C_sur_* = (1.06 ± 0.04)∙10^21^ cm^−1^ at the surface of the sample. The preparation conditions have been set so that the Ti concentration gives rise to a refractive index profile with surface refractive index jumps Δ*n_e_* = (1.12 ± 0.03)∙10^−2^ for the extraordinary index and Δ*n_o_* = (0.66 ± 0.02)∙10^−2^ for the ordinary one, respectively [46]. These values are optimized to confine inside the waveguides light beams with wavelengths ranging from visible to infrared. Finally, it is important to underline that this microfabrication strategy allows the realization of multiple waveguides on the same LN substrate, as shown in Figure 2.

#### 2.1.2. Microfabrication of the Channel

The sample with the diffused multiple waveguides is engraved in the middle with an orthogonal microchannel by means of a self-polishing precision saw DISCO DAD 3350 (Disco Corporation, Tokyo, Japan), as shown in Figure 2a.

The saw machine is equipped with a polymeric blade coated with diamond particles that, as will be demonstrated later, guarantees a good quality for both the channel surfaces and the external sides of the device. Notably, the first is important for the light transmission between the two branches of the waveguide across the channel (see Figure 2b), while the second is important for achieving a proper butt-coupling in the waveguide without need of an extra polishing step. Therefore, the same procedure is also used to cut the lithium niobate samples from the wafer. Considering the importance of the channel surface quality for the working principle of the device, a comparison between the microchannel engraved by using two saws with different thicknesses (50 and 200 µm) will be discussed in the Results and Discussion paragraphs. Microchannels with a length of 2 cm are realized with both blades, and two different nominal cross-sections are obtained: 200 × 100 µm^2^ for the 200 µm-thick blade and 50 × 100 µm^2^ for the 50 µm. For both blades, the best surface quality is achieved with a rotation speed of 10,000 rpm and continuous lubrication with water. The device is then sealed by a glass top cover equipped with inlet/outlet using a UV curable glue (NOA68, by Norland Optical Adhesive Inc., Cranbury, NY, USA), as shown in Figure 2c. In detail, both glass and lithium niobate are rinsed with acetone and distilled water. Successively, the two are kept in tight contact and the glue is gently distributed all around using a syringe (<1 µL drop at each side of the bonding surface), avoiding the part in intimate contact with the microfluidic channel feature. In this way, the glue flows inside the thin air gap between the two substrates due to the capillary pressure. According to Langelier et al. [38], the abrupt changes in hydraulic resistance at the edge of the channel confine the advancing glue front, thus avoiding the risk of flooding the microfluidic channel [38]. Finally, the entire device is exposed to UV light for 7 min [38], obtaining the final device (see Figure 2d).

### 2.2. Opto-Microfluidic Setup

**Figure 3:**
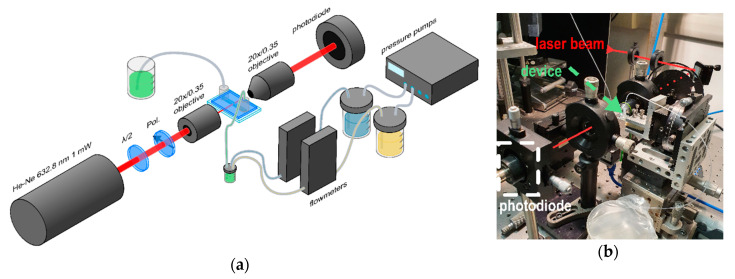
Opto-microfluidic setup allows controlling the flow of the liquid into the microchannel and the coupling of the laser beam in the waveguide. (**a**) Scheme of the setup used for the waveguide characterization and for the proof of concept experiment, in which the LN device is used as a pH meter. In the latter, two different solutions (blue and yellow in the scheme) presenting different pH values are independently flown and mixed into a common container. The resulting mixture (green) is driven into the microfluidic device. (**b**) Picture of the optical part of the experimental setup.

The setup used for the device characterization and the proof of concept experiment is reported in Figure 3; it is composed of an optical and a microfluidic part. The optical part consists of an He-Ne CW laser (632.8 nm 1 mW by Coherent Inc., Santa Clara, CA, USA) which is butt-coupled to the input waveguide by an objective lens (Achromat-20X/0.35 WD 0.17 by Olympus, Tokyo, Japan); similarly, an identical objective is employed to collect the light from the output waveguide after the transmission across the channel. The polarization of the light source is controlled by a half waveplate and a polarizer. The light signal collected by the second objective is driven to a photodiode and amplified by a lock-in amplifier (SR830 by Stanford Research Systems, Sunnyvale, CA, USA), which controls also the optical chopper located after the polarizer [47]. It is worth mentioning that fiber pigtailed coupling can be easily implemented instead of objective lenses.

The fluidic part consists of a pumping system used to control the flowrates of two liquids, which are an acidic and a basic solution, as will be detailed later. Both solutions are injected into the same container, which acts as a mixer, by a pressure pump (OB1 MK3 by Elveflow, Paris, France) in feedback with two coupled flowmeters (BFS Coriolis by Bronkhorst High-Tech BV, AK Ruurlo, The Netherlands). The mixed solution is finally pumped inside the microchannel of the device, where the optical transmission from the waveguides is modulated by the liquid absorbance. This simple configuration allows easily tuning the concentration ratio between the two initial solutions in the microfluidic device by adjusting the ratio between the respective flowrates.

### 2.3. pH Solutions

For the proof of concept experiment in which the device is used as a pH meter, two initial solutions are prepared presenting pHs of 2.7 and 10.5, thus showing an acid and a basic character, respectively. They are prepared by mixtures of ultrapure water (by Millipore, Burlington, MA, USA) with hydrochloric acid (HCl 37%, Sigma Aldrich Inc., St. Louis, MO, USA) and sodium hydroxide (NaOH ≥ 97.0%, Sigma Aldrich Inc., St. Louis, MO, USA), respectively. The pH values are characterized by a pH meter (HI-8521 by Hanna Instruments, Woonsocket, RI, USA). Notably, in both initial solutions, Bromothymol Blue (C_27_H_27_Br_2_NaO_5_S, Sigma Aldrich Inc., St. Louis, MO, USA) is added as a pH indicator at a concentration of 120 µM. This guarantees that, after the mixing of the initial solutions, the pH indicator is always present in the same concentration, independently from the mixture ratio fixed by the flowrates. As will be discussed later, the optimal concentration value for the pH indicator is obtained by the UV/visible spectral analysis presented in the next section, obtained by the spectrophotometer (V-670 UV-VIS by Jasco Europe, Cremella, Italy).

## 3. Results and Discussions

### 3.1. Device Design: Flexibility and Characterization

The coupling between the waveguides and the microchannel has been fully characterized, demonstrating the high efficiency and flexibility of this optofluidic system. As a matter of fact, the width and depth of the channel should be controlled at will, while the optical probe must be demonstrated to be versatile in terms of the optical properties of the light source, such as wavelength and polarization, in order to achieve a high-performance sensor. Finally, the quality of the channel surfaces can influence the light transmission across the fluidic circuit, therefore the roughness of the surfaces has to be properly optimized in order to avoid scattering losses compromising the optical sensing process. These aspects will be discussed in the next two sections.

#### 3.1.1. Waveguides Characterization

**Figure 4:**
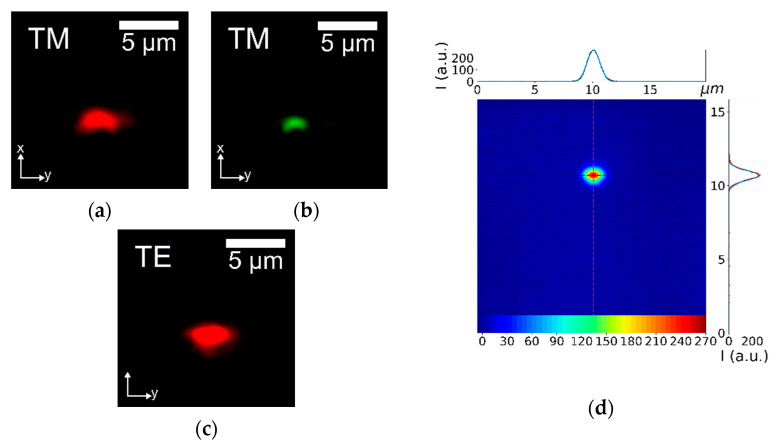
Examples of near-field CCD images of the output of the Ti-undiffused waveguide excited with different light sources. (**a**,**b**) report the output of a waveguide which is coupled with a laser beam at 632.8 and 533 nm, respectively. Both the first two figures represent Transverse Magnetic (TM) propagation modes, whereas (**c**) refers to the same waveguide of (**a**), but supporting a Transverse Electric (TE) propagation mode. (**d**) Beam profiling analysis of the output light from a waveguide excited by a laser beam in TM polarization at 632.8 nm. The gaussian profiles on the top and on the right represent the intensity profile of the outcoming beam along the y-axis and the x-axis of the LN device, respectively.

The waveguides represent the optical probe of the final microfluidic device. Therefore, the flexibility of the sensor in terms of light wavelength and polarization is strictly related to the capability of realizing waveguides able to guide light beams with different properties. In order to test such versatility, light sources at 532 and 632.8 nm are employed, as well as light polarizations Transverse Electric (TE) and Transverse Magnetic (TM). In detail, Figure 4a,b show the transmitted modes corresponding to laser beams at 632.8 and 533 nm in TE polarization, respectively, while Figure 4c is the analog of Figure 4a but with TM polarization. These images are obtained with the near-field technique using a CCD camera (LaserCam-HR II by Coherent Inc., Santa Clara, CA, USA), and they represent the intensity shape of the light exiting from the output waveguide.

As is possible to notice in Figure 4a,b, the Ti:LN waveguide allows an efficient propagation of light beams at both wavelengths; similarly, Figure 4a–c clearly demonstrate that the light polarization does not significantly change the transmission property of the waveguide, neither in terms of the beam shape nor its intensity. This feature can be relevant for specific applications, where the light polarization could play a key role, such as applications involving Liquid Crystal [33,48]. Notably, all the beam outputs in Figure 4 show a monomodal behavior, which is ideal for realizing in the future a fiber pigtailing of the waveguides, thus assuring the complete portability of the final devices. Finally, a profile analysis of the guided beams is performed by means of a beam profiler (LaserCam-HR II by Coherent Inc., Santa Clara, CA, USA), as reported in Figure 4d. Both profiles of the beam mode along the two main directions of the crystal (i.e., the x-axis and y-axis) are well described by a gaussian function, as illustrated in the insets of Figure 4d; the latter confirms the possibility of realizing an optimal pigtailing with the Ti:LN waveguides, which is expected to guarantee even an higher coupling efficiency [49]. Finally, another important feature of an optical waveguide is its extinction coefficient. The propagation losses has been measured by the cut-back technique in the visible range for several waveguides, showing an average extinction coefficient of 3 ± 1 dB cm^−1^, which is in good agreement with the standard values [50]. This efficient transmission allows sensing applications even for low amounts of light source power, as in the presented experiment (1 mW). Notably, this feature is important in order to avoid unwanted heating effects on the liquid due to the laser light, which could strongly influence the final purpose of the device, such as pH sensing.

#### 3.1.2. Microchannel Quality Fabrication

The key feature for an efficient coupling of waveguides with the microchannel is the quality of the channel lateral surfaces. In particular, the upper edges of the channel (i.e., the location at which the waveguide intersects the channel) should be sharp and without the presence of scratches. Additionally, a high roughness of the channel surfaces can compromise the light propagation due to scattering losses. The roughness of the channel bottom and walls has been already characterized in [24], showing average values of 8.5 ± 0.9 nm and 23 ± 7 nm, respectively, for different crystallographic orientations and dopant concentrations [51]. In this work, we tested and compared the results obtained using two different blades with widths of 50 and 200 µm.

**Figure 5:**
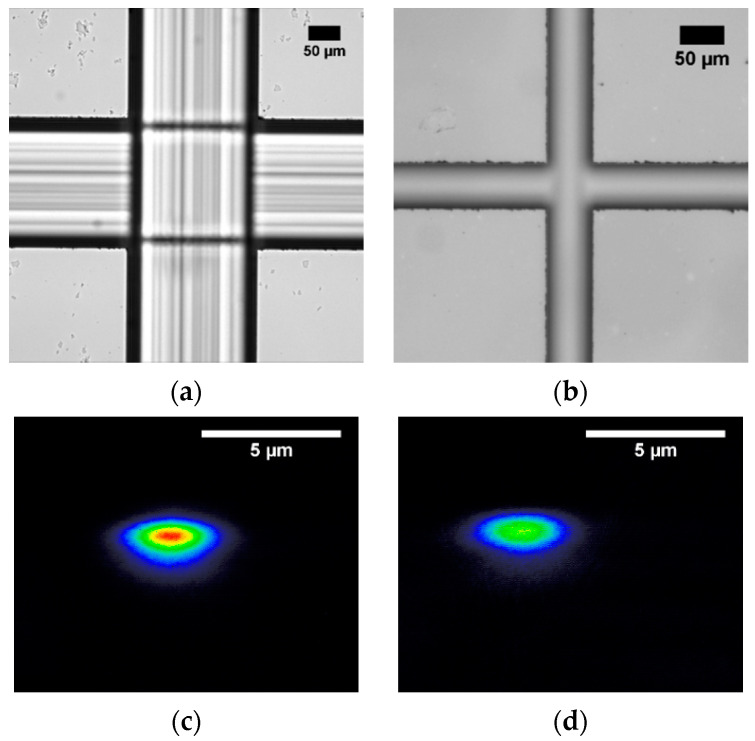
Optical microscope images of microfluidic channels realized with different blade thicknesses. (**a**,**b**) show the channels realized with 200 and 50 µm-thick blades, respectively. (**c**,**d**) report examples of the output waveguides captured by the near-field technique, referring respectively to channels obtained by with 200 and 50 µm-thick blades. Both structures do not evidence any differences between the two crystallographic directions.

Figure 5 reports two optical microscope images of the channels realized using the two different blades. The cross shape is chosen to test the channel quality in two crystallographic directions, which show similar results in terms of scratches. The bottom surfaces of the channel present in both cases (Figure 5a,b) the same longitudinal pattern, characterized by distinctive stripes in the direction of the blade rotation. This roughness does not influence the light transmission across the channel [24]. On the contrary, the scratches on the upper edges of the channel can drastically deny the light transmission; in detail, the channel realized with the 50 µm-thick blade (Figure 5b) clearly presents more scratches on the upper edge compared to the 200 µm one (Figure 5a). In order to test the influence of these scratches on the coupling between the waveguides and the fluidic channel, the light transmission of more than 10 waveguides across the channel is tested. In the 200 µm case, all the waveguides transmit light due to the low amount of scratches (see Figure 5c). For the 50 µm one, we observed that only 1 out of 10 denied transmission, due to the presence of scratches, while the others waveguides work properly (see Figure 5d). Therefore, even if the presence of surface defects limits the coupling between the waveguides and the channel, the capability of producing several waveguides on the same device during the microfabrication allows selecting the more appropriate one in order to achieve a sufficient optical coupling. Since the cutting parameters used for the two cuts are the same, the poor quality of the 50 µm channel could be probably due to the transversal oscillation of the blade during the high rotation speed. This explanation is also confirmed by the comparison between the real widths of the obtained channels and the nominal ones. As reported in Table 1, while using the blade of 200 µm leads to a channel width compatible with the blade size (i.e., the nominal width of the channel), the blade of 50 µm produces channels larger than about 10 µm. This discrepancy can be observed only in the widths and not in the depths (Table 1), confirming the presence of an eventual oscillation only in the transverse direction.

### 3.2. pH Sensing Based on Bromothymol Blue Absorbance

Once both the waveguides and the quality of the device surfaces are characterized, the proof of concept of the optical pH sensing by the LN device is presented in the following. Firstly, the spectral analysis of Bromothymol Blue is studied to give a prediction of the optical response of the device. Secondly, the concentration of the pH indicator used for the opto-microfluidic measurements is investigated. Finally, the optical response of the opto-microfluidic LN device is presented and discussed.

#### 3.2.1. Bromothymol Blue Spectral Analysis

**Figure 6:**
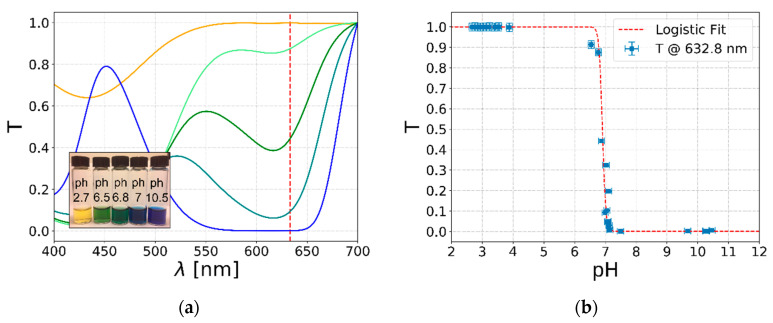
Spectral analysis for the Bromothymol Blue pH indicator. (**a**) The graph shows 5 spectra of solutions with different pHs; the line colors are related to the color assumed by the corresponding solution, referring to the inset. The red dashed line evidences 632.8 nm, which is the wavelength used for the pH sensing by the integrated LN device. (**b**) The graph reports the transmission at 632.8 nm for solutions at different pHs. At the transition, the transmission values present a sigmoidal behavior, which is confirmed by the interpolation of the data with an exponential logistic function (see Equation (1)).

Optical pH sensing based on absorbance relies on the spectral transition of the pH indicator, due to a change in the pH of the analyte solution. In order to obtain a reliable sensing by the LN device, the relationship between the Bromothymol Blue absorbance variation and the pH is studied considering the transmittance spectra of 16 solutions with pHs between 2.7 and 10.5. By way of example, Figure 6a shows the spectra of five solutions. Since the pH measurement is based on the transmission differences of Bromothymol Blue indicator between solutions at different pHs, the plots show that the highest variation sensitivity for Bromothymol Blue is obtained for wavelengths in the range of 600–650 nm. For this reason, the LN chip sensing experiments are performed with a 632.8 nm light source (see the red vertical line in Figure 6a). Then, the transmittance (*T*) transition depending on the pH is characterized at this wavelength in Figure 6b, where the blue points are the average of 10 repeated spectra and the average of five independent measurements of the pH. The graph shows a well-distinguished non-linear sigmoidal behavior which is the combination of the Henderson–Hasselbach equation and the optical response of the Bromothymol Blue. In good approximation, this behavior can be described by a logistic function [52]:

(1) $$T=\frac{1}{1+e^{-\left(pH-pH_{c}\right)/\Delta }},$$

where *pH_c_* represents the equivalence points for Bromothymol Blue, and Δ is the *pH* range in which the transition occurs. The interpolation of the experimental data with Equation (1) leads to *pH_c_ =* 6.9 ± 0.1 and Δ = 0.06 ± 0.01. These results are in good agreement with the literature values for an equivalence point at 7.1 and a range between 6.2 and 7.6 obtained with a different wavelength in [53].

#### 3.2.2. Bromothymol Blue Concentration

**Figure 7:**
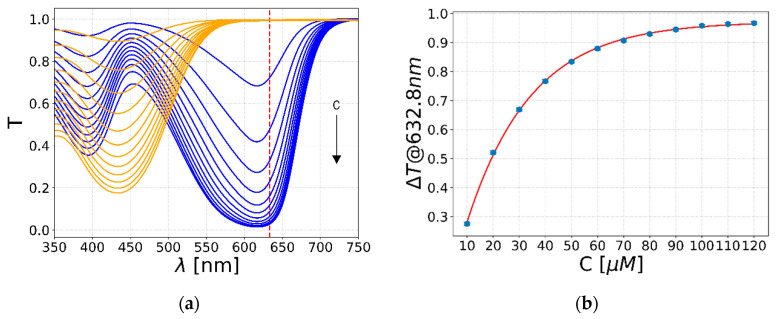
Concentration spectral analysis of Bromothymol Blue. (**a**) The graph reports the transmission spectra in the visible range; the blue lines are the spectra of the 12 solutions with *6* pH, whereas the orange ones refer to solutions with 7.6 pH. The arrow indicates the increasing concentration of the pH indicator in the two solutions. (**b**) The transmittance difference Δ*T* between the two solutions at 632.8 nm is plotted in the function of Bromothymol Blue concentration.

Another key parameter to be characterized before the pH measurements with the LN chip is the optimal concentration of Bromothymol Blue inside the analyte solutions, which must be chosen in order to maximize the optical response of the system. To this aim, a spectral analysis of two solutions with pHs of 6 (orange lines) and 7.6 (blue lines) with 12 different pH indicator concentrations between 10 and 120 µM is performed (see Figure 7a). The pH values are chosen to lie on the two sides of the transition of the Bromothymol Blue (Figure 6b). As shown by the graph, higher concentrations of pH indicator lead to a lower transmittance in both acid and basic solutions across all the visible range, as expected by the Lambert–Beer law:

(2) $$T=e^{-A}=e^{-\varepsilon \;l\;C},$$

where *A* is the absorbance of the solution, ε is the molar attenuation of the Bromothymol Blue at the investigated pH and wavelength, *C* is the indicator concentration, and *l* is the optical path length. The transmittance difference (Δ*T*) between the two solutions at 632.8 nm is plotted in Figure 7b against the indicator concentration. Since *l* and *ε* do not depend on C, the transmittance difference shows an exponential trend with respect to the Bromothymol Blue amount. Therefore, the optimal indicator concentration for the purpose of this work is 120 µM, which leads to a high transmittance difference, keeping the same pH of the analyte solution.

In fact, due to the exponential trend of Figure 7b, an increase in concentration will not improve drastically the transmittance difference, whereas, on the other hand, it could lead to an undesired change in the pH of the solution due to the high concentration of the pH indicator. Furthermore, the predicted transmittance difference for the LN chip at 120 µM of indicator concentration is Δ*T* = 0.12 ± 0.01, which is sufficient to distinguish the pH of the liquid inside the channel by means of the optical response of the waveguide. This estimation is obtained from Equation (2), considering that the spectral analysis is performed with a standard cuvette with *l* = 1 cm, whereas the LN chip of this work has *l =* 200 µm. The comparison with the actual results and this prediction will be discussed in the next paragraph.

#### 3.2.3. Integrated Optical pH Sensing Experiments

The experimental validation of the integrated optical pH sensing consists of the simultaneous injection in the mixing container of the two initial solutions, whose flowrates can be properly tuned to obtain inside the microchannel an analyte solution with a tunable pH. At the same time, the transmittance of this analyte solution is measured by the optical response from the waveguide.

The system is tested by flowing 11 consecutive and different mixtures every 3 min, following the flowrates reported in Table 2. Specifically, every 3 min (time interval) the flowrates of the two solutions with pHs of 2.7 *Q_acid_* (HCl solution) and 10.5 *Q_basic_* (NaOH solution) are varied to progressively change the pH of the analyte solution. Two independent measurements are performed: the analyte solutions injected in the microchannel vary from acid to basic, and vice versa. The measurement starting from the acid (basic) solution will be called HCl neutralization (NaOH neutralization). The same flowrate protocol with reversed time intervals is used for both measurements. During all the 33 min, the optical transmission signal from the waveguide is recorded with a frequency of 1 Hz. The latter is chosen in order to acquire enough data points to smoothly describe the transition region. Notably, the acquisition frequency is not limited by the optical part of the system, while by the parameter we have chosen for the electronic data acquisition—the used lock-in amplifier (SR830, Stanford Research System, Sunnyvale, CA, USA)—can achieve up to 512 Hz. This value can be easily improved by changing the performance of the electronic system.

**Figure 8:**
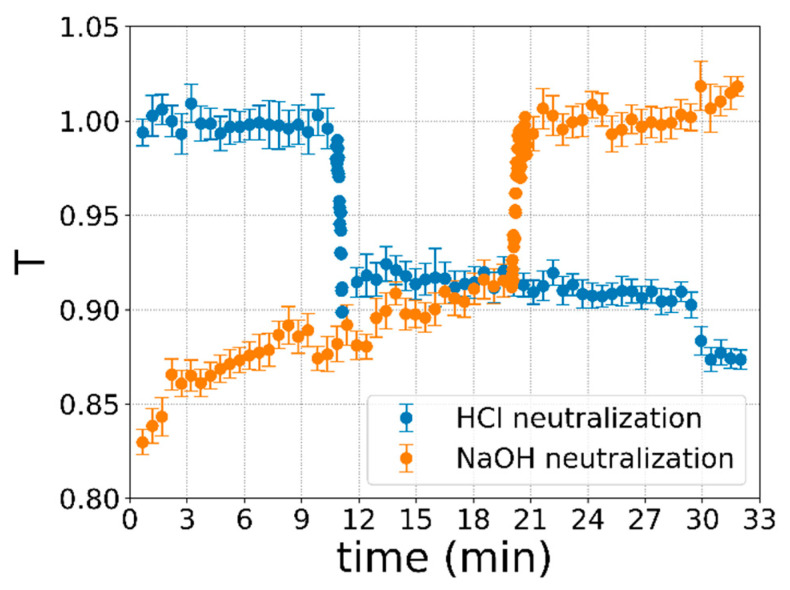
Optical transmission responses from the waveguide on the optofluidic chip, obtained using the flowrate recipes of Table 2. The orange data correspond to the neutralization process of NaOH, whereas the blue ones correspond to the HCl. For the purpose of clarity, the data are smoothed considering an average every 20 s and the related standard deviation as the error, except for the abrupt transition in the correspondence of the pH indicator equivalence point.

Figure 8 shows the results of the optical transmittance measurements performed with the optofluidic LN-based chip, normalized to the average of the higher plateau of the signal (Figure 6b). The abrupt change in light transmission clearly detects the moment at which the analyte solution in the channel has a pH equal to the pH transition. Both measurements show symmetry with respect to the temporal axis, which is proportional to the concentration of HCl and NaOH injected into the microchannel, which, as expected, takes place in an opposite way in the two measurements (Table 2). The drop and rise in the transmission signal (Δ*T*) occurring during all the measurements are derived considering the differences between the average values of T of the first and last interval of the protocol reported in Table 2. They result in Δ*T_NaOH_* = 0.14 ± 0.01 and Δ*T_HCl_* = 0.10 ± 0.01 for the neutralization of the NaOH and HCl solution, respectively. Both results are in good agreement with the prediction made in the previous section of Δ*T* = 0.12 ± 0.01. The steepness and low dispersion of the experimental points in the transition interval confirms the high sensitivity of the optical waveguide. Differently, the higher data dispersion outside the transition interval is attributed to the low sensitivity of the pH indicator spectral variation at the chosen laser wavelength (632.8 nm).

**Figure 9:**
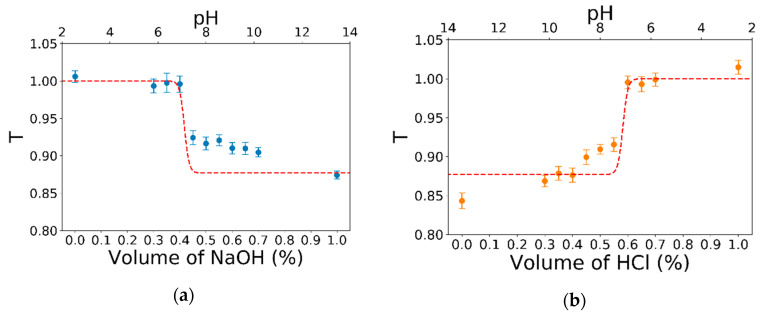
Comparison between the optical transmission responses obtained with the waveguide and the spectrophotometer analysis for the HCl neutralization (**a**) and for the NaOH neutralization (**b**). The blue and orange points representing each 3 min of data acquisition are plotted against the concentration of neutralizing species in the bottom axis (i.e., NaOH for HCl neutralization and HCl for the NaOH one). The red dashed lines, representing the prediction obtained from fits in Figure 6b by Equation (2) with *l* = 200 µm, are plotted against the pH values in the top axis. The alignment between the two horizontal axes has been made considering the same center of the transition event.

The optical pH sensing is finally achieved by the correlation between the measurements of optical transmission in Figure 8 and the pH of the injected analyte solutions. Such correlation is reported in Figure 9 for both the measurements of HCl (a) and NaOH neutralization (b). Both plots consider an average of only the central 60 s of each 3 min time interval, where the specific concentration of HCl and NaOH is stabilized in order to limit any mixing problems. The relation with the pH can be obtained by the prediction analysis performed in the previous section. In detail, the transmission T depending on the pH described by the logistic interpolation of Equation (1) in Figure 6b is rescaled considering the *l =* 200 µm using the Labert–Beer law (Equation (2)). The results of such a prediction are reported as a dashed red line in both graphs of Figure 9, where the alignment between the pH axis and concentration is made by considering Δ*T* in the same x-axis range and the same center of transition for the x-axis, pH, and volume fraction. This final correlation between the pH values and the transmitted light confirms the feasibility of using the integrated LN system as a sensor for the pH value of an analyte solution. In particular, for *T* > 0.95 the solution has a pH < 6.8, whereas for *T* < 0.95 the solution has a pH > 7.3. Additionally, in the pH range with the highest sensitivity for the pH indicator, the pH can be measured with a sensitivity of 0.04. Indeed, the transmittance T changes 0.10 ± 0.01 over a variation in the pH of only 0.4 ± 0.1 for analyte solutions with a pH between 6.4 and 7.2.

The limit of detection (LOD) of the presented sensor is evaluated as the smallest Δ*T* that can be measured. According to the uncertainty of the data reported in Figure 9, this value has been estimated as the highest standard deviation obtained for a single point in the graph, which is found to be 0.013. The minimum transmission difference to distinguish two points *T* is considered as five times this standard deviation value. Therefore, the LOD in terms of the Bromothymol Blue concentration is found to be 1.58 ± 0.01 µM (exploiting the conversion in Figure 7a). Notably, the result improves the performances achieved with the polymeric waveguides [13], which showed an LOD 18 times higher than the presented system using the same pH indicator. In addition, the LOD could be further improved by the pigtailing of the optical fiber [49], which is expected to reduce the background noise of the transmission signal *T*. Finally, the versatility of the LN:Ti optical waveguide can be exploited in combination with other pH indicators, also based on fluorescent measurements. Additionally, the filters needed for fluorescent dye application can be integrated directly in the LN substrate using Bragg’s grating [54].

## 4. Conclusions

We have presented an optofluidic device completely realized in a monolithic lithium niobate crystal, where optical and microfluidic components have been properly integrated. In detail, we combined optical waveguides, realized by the thermal diffusion of titanium thin stripes on LN, with a microfluidic channel mechanically engraved onto the same substrates by means of a self-polishing saw. This microfabrication strategy allows achieving a perfect alignment across the microchannel of the input and the output waveguides, overcoming the typical limitations typically occurring in the optical fiber embedding process [8,9,10,11,12,55]. Then, the coupling between the optical and fluidic components of the device has been demonstrated to efficiently work for different channel sizes—i.e., 50 and 200 µm. Therefore, the proposed optofluidic device can be easily integrated within more complex fluidic geometries, which can be obtained in LN substrate by other fabrication techniques, such as laser ablation [24,33] or Focused Ion Beam [56]. Additionally, the Ti:LN waveguides have been demonstrated to be able to guide laser beams with different polarizations and wavelengths in the visible range—i.e., 533 and 632 nm. These results highlight the versatility of these optical probes, which is further enhanced by the high parallelization capability offered by the waveguide preparation process employed in this study, which allows realizing one waveguide every 10 µm.

Finally, as a further demonstration of its quality and efficiency, the proposed optofluidic device is exploited for the pH optical sensing of an analyte solution. The light transmitted by the waveguides is used as an optical probe able to monitor in real-time any variation in the transmittance of the investigated solution, caused by a change in its pH value. In detail, Bromothymol Blue is employed as a pH indicator, which presents an equivalence point close to pH = 7. The LN-based device has been tested by continuously mixing two initial solutions with acid and basic characters, and by the real-time monitoring of the transmitted light while the pH varied from 2.7 to 10.5 and vice versa. Both results presented a transmittance change in agreement with the one predicted by the Bromothymol Blue, allowing us to characterize a sensitivity of 0.04 pH at the transition interval and a limit of detection of 1.58 ± 0.01 µM. These results demonstrate an improvement of almost 18 times compared to the polymeric waveguide systems [13]. Moreover, the pH transition is found to be in perfect agreement with the expected value, suggesting that the laser light does not influence the temperature of the liquid flowing in the channel and, thus, the pH measurement.

Obviously, since the Ti:LN waveguides can guide beams with different wavelengths, this sensor can be used with pH indicators other than Bromothymol Blue, increasing the range of pH values that can be investigated and, consequently, the range of applications of the device, including the use of fluorescent-based dyes. Furthermore, the device offers the key feature of real-time monitoring essential for an eventual feed-back system, where a solution with a desired pH can be easily obtained, in combination with a flowrate controller [57].

Besides the results mentioned above for pH sensing, we believe that the presented optofluidic device based on lithium niobate is extremely promising in the field of LOC systems, thanks to its high degree of versatility and integrability with other analysis components. In fact, the possibility of using beams with different wavelengths for the optical analysis, as well as the flexibility in realizing fluidic channels, make the proposed device suitable to be employed in different and more complex applications, such as the characterization of droplet-based systems or biological samples.

## Figures and Tables

**Table 1:** Results of the profile analysis carried out on 10 different channels, in different positions inside them. More than 10 profiles are considered for each channel, using the profilometer P-10 (KLA Tencor, CA, USA). The table reports the difference between the average values and the nominal ones.

Blade Thickness	Nominal Width	Resulting Width	Nominal Depth	Resulting Depth
50 µm	50 µm	61 ± 1 µm	100 µm	98 ± 1 µm
200 µm	200 µm	201 ± 1 µm	100 µm	99 ± 1 µm

**Table 2:** Example of the flowrate protocol for one measurement, where every 3 min the analyte solution is obtained from a different combination of two solutions, acid and basic ones. *Q_acid_* refers to the flowrate of the acid solution, whereas *Q_basic_* refers to that of the basic one. The same protocol is used for another measurement, but with the interval reversed.

Time Interval (min)	*Q_basic_* (µL min^−1^)	*Q_acid_* (µL min^−1^)
0–3	100	0
3–6	70	30
6–9	65	35
9–12	60	40
12–15	55	45
15–18	50	50
18–21	45	55
21–24	40	60
24–27	35	65
27–30	30	70
30–33	0	100
